# Maternal knowledge and clinically relevant care gaps in preterm infant care in Gaza, Palestine: a cross-sectional study

**DOI:** 10.1186/s12887-026-07188-5

**Published:** 2026-06-23

**Authors:** Mohammed Abdeljawad, Ahmed Najim, Hamza Abdeljawad, Josie Rodgers, Reham Almukbel, Kinan Mokbel

**Affiliations:** 1 Faculty of Health Professions, Al-Quds University, Abu Dis, Palestine; 2 Department of Nursing, Faculty of Applied Medical Sciences, Al-Azhar University, Gaza, Palestine; 3 Department of Health and Care Professions, Faculty of Health and Life Sciences, University of Exeter, Exeter, UK; 4 Royal Devon University Healthcare NHS Foundation Trust, Exeter, UK; 5 Faculty of Medicine, Al-Sham Private University, Damascus, Syria

**Keywords:** Premature infant, Maternal knowledge, Neonatal care, Antenatal care, Feeding, Phototherapy

## Abstract

**Background:**

Mothers of premature infants need practical knowledge to support feeding, thermal care, infection prevention, phototherapy safety and discharge preparation. Evidence on domain-specific maternal knowledge in resource-constrained neonatal settings remains limited. This study assessed maternal knowledge of premature infant care in Gaza and explored clinically relevant item-level knowledge gaps to inform future education priorities.

**Methods:**

A cross-sectional survey was conducted among 170 mothers of premature infants admitted to neonatal departments in four government hospitals in the Gaza Strip. A 30-item interviewer-administered questionnaire assessed knowledge across thermoregulation, feeding, phototherapy, and infection and skin care. Descriptive, bivariate and adjusted regression analyses were conducted, with exploratory item-level analyses used to identify clinically relevant knowledge gaps.

**Results:**

Overall knowledge was moderate, with a mean score of 64.1% (SD = 22.3), but 53 mothers (31.2%) had poor knowledge. Knowledge differed across domains (*p* < 0.001), with feeding the weakest domain (53.6%) and infection and skin care the highest by observed percentage score (73.8%). In the parsimonious adjusted model, not receiving specialist premature-care antenatal follow-up was associated with lower odds of higher knowledge (adjusted OR = 0.44, 95% CI 0.20–0.97, *p* = 0.042). Item-level findings showed clinically relevant gaps in feeding tolerance, nil per os (NPO) management, nasogastric tube (NGT) feeding, phototherapy safety, handling practices and umbilical cord care.

**Conclusions:**

Although overall maternal knowledge was moderate, this masked clinically important gaps in practical caregiving domains, particularly feeding, phototherapy safety, handling practices and umbilical cord care. Given the cross-sectional design, findings should not be interpreted causally. These findings may inform the development and future evaluation of domain-specific education tools for antenatal counselling, bedside teaching and discharge preparation in similar constrained neonatal settings.

**Supplementary Information:**

The online version contains supplementary material available at https://doi.org/10.1186/s12887-026-07188-5.

## Background

Preterm birth, defined by the World Health Organization (WHO) as birth before 37 completed weeks of gestation, remains a major contributor to neonatal morbidity and mortality worldwide [1]. Millions of infants are born preterm each year, with substantial variation between countries and settings [2]. Preterm birth complications are also an important contributor to child mortality [3]. Premature infants are vulnerable to longer-term health and developmental consequences, including neurodevelopmental impairment, chronic lung disease and later cardiometabolic or renal risk [4]. Very preterm infants are also at increased risk of sensory impairment, including hearing loss [5]. Large cohort data show substantial morbidity and mortality among extremely preterm infants, including respiratory distress, necrotising enterocolitis and sepsis [6]. Prematurity also carries longer-term health and educational costs [7], while severe intraventricular haemorrhage is also an important adverse outcome in preterm-infant research and clinical trials [8]. 

Prematurity is particularly important in Gaza, where WHO has described childbirth as especially dangerous because mothers and newborns face overburdened hospitals and limited healthcare support before and after birth [9]. UNICEF has reported that 23% of births in Gaza are preterm, with approximately 10,000 neonates requiring neonatal intensive care transfer annually [10]. This burden occurs alongside poverty and conflict-related maternal and neonatal health challenges [11, 12]. Humanitarian-system disruption may also limit mothers’ opportunities to receive, understand and retain premature-care education, as staff workload, disrupted antenatal follow-up and pressure on hospital services may reduce counselling time and discharge preparation. Identifying the weakest caregiving knowledge areas is therefore important so that limited education time can be directed towards clinically relevant gaps.

The care of premature infants requires informed maternal and family participation. WHO guidance emphasises family involvement, parental support and education as part of routine care for preterm or low-birth-weight infants [13]. Maternal knowledge is clinically relevant because feeding support, thermal protection, hand hygiene, umbilical cord care, recognition of danger signs and engagement with treatments such as phototherapy all depend partly on day-to-day caregiving. Knowledge alone does not ensure safe care, but it may support understanding of clinical instructions, bedside participation and care after discharge, particularly in resource-constrained neonatal settings where staff time and discharge preparation may be limited. Practical and emotional support from neonatal nurses can also help mothers participate more confidently in care [14]. 

Because maternal knowledge of premature infant care is most useful when linked to practical caregiving tasks, this study focused on four parent-facing domains: feeding, thermoregulation, phototherapy, and infection and skin care. These domains cover feeding readiness, nil per os (NPO) and nasogastric tube (NGT) feeding, heat-loss prevention, phototherapy monitoring and protection, hand hygiene, umbilical cord care and infection monitoring. Existing studies often report overall knowledge scores, with less attention to domain-specific gaps, especially in resource-constrained and conflict-affected neonatal settings. This study therefore assessed maternal knowledge across these four domains, examined associated factors and explored item-level clinically relevant gaps to inform future antenatal counselling, neonatal-unit parent education and discharge preparation.

## Methods

### Study design and setting

This cross-sectional study was conducted between March 2022 and March 2023 in the neonatal departments of four government hospitals in the Gaza Strip, Palestine: Al-Shifa Medical Complex, Al-Aqsa Hospital, Nasser Hospital and Emirati Hospital. These hospitals provide government neonatal care, including neonatal intensive care services, and receive premature infants from across the Gaza Strip. They were selected because they are major governmental referral sites for premature infants and together represented the main hospital settings from which eligible mothers could be recruited during the study period. Estimated annual premature-infant admissions varied across hospitals and were used for proportional sample allocation (Additional file 1, Table S1).

### Study population and sampling

The target population comprised mothers who had delivered a premature infant admitted to one of the selected neonatal units during data collection. Sample size was calculated using Epi Info StatCalc for a finite population, based on estimated two-month premature-infant admissions derived from annual hospital admission estimates. As no prior estimate of adequate maternal knowledge was available, a conservative expected proportion of 50%, 95% confidence level and 5% margin of error were used. This produced a required sample of 172 mothers, allocated proportionally across hospitals (Additional file 1, Table S1).

Consecutive sampling was used until the required sample for each hospital was reached. Eligible mothers were approached in hospital during their infant’s neonatal admission, after study procedures had been explained and written informed consent obtained. Mothers were eligible if they had delivered a premature infant admitted to one of the selected hospitals and provided written informed consent; they were excluded if their infant was not present in the neonatal unit at interview. Interviews were conducted during admission, but their timing in relation to counselling or discharge preparation was not standardised. Non-participant characteristics were not available for comparison with participants, and the pragmatic sampling approach may limit representativeness.

### Questionnaire development, translation and validation

Data were collected using a researcher-designed, interviewer-administered questionnaire developed from literature on premature infant care, maternal knowledge and parent-facing neonatal care practices. The items were not directly adapted from a single validated instrument, but were developed to reflect practical premature infant care topics relevant to the study setting. The questionnaire covered sociodemographic and obstetric characteristics, maternal health-service exposure, information sources and maternal knowledge. Within the maternal health-service section, specialist premature-care antenatal follow-up was defined as mother-reported antenatal follow-up specifically related to premature infant care, beyond routine antenatal visits or general prenatal examination. It was assessed as a yes/no item, so details of content, provider type, timing, duration and frequency were not captured. The full English and Arabic versions are provided in Additional file 1. The questionnaire was developed in English and translated into Arabic. Both versions were reviewed for clarity, cultural appropriateness and consistency of meaning. Formal back-translation records were not available for reporting and this is acknowledged as a limitation.

The knowledge section included 30 dichotomously scored items across four conceptually generated practical care domains: thermoregulation, feeding, phototherapy, and infection and skin care. These domains were selected because they represent common parent-facing areas of premature infant care during admission and around discharge. Seven specialists reviewed the domains and items for relevance, clarity, comprehensiveness, cultural appropriateness and language consistency and their comments were used to refine wording and content. The domains were not statistically derived using factor analysis, and the specialist review was qualitative rather than a formally scored content-validity exercise; therefore, item-level and scale-level content-validity indices were not available for reporting.

A pilot study with 20 mothers assessed feasibility, clarity, administration time and internal consistency. The pilot did not identify the need for substantive changes to item wording, structure or administration procedures; therefore, no post-pilot modifications were made, and pilot participants were included in the final analysis.

### Knowledge scoring and statistical analysis

Each correct knowledge response was scored 1 and each incorrect response 0. The overall score was calculated as the percentage of correct responses across the 30 items, with each item weighted equally; domain scores were calculated as percentage correct within each domain. Overall knowledge was categorised as poor (< 50%), good (50–70%, inclusive) or excellent (> 70%), following Al-Mukhtar and Abdulghani [15], to support comparison with previous work. These categories were treated as pragmatic descriptive thresholds rather than validated clinical cut-offs. Because categorisation may reduce information and statistical power, the continuous overall knowledge score was also analysed in sensitivity analysis. Alternative thresholds were not examined because no validated cut-offs were available for this population.

Internal consistency was assessed using Cronbach’s alpha, interpreted as equivalent to Kuder-Richardson 20 because items were dichotomously scored. Item performance was examined using correct-response proportions across all 30 items. For the infection and skin care domain, which had the lowest internal consistency, corrected item-total correlations and alpha-if-item-deleted values were also examined. Items were not removed post hoc because the aim was descriptive rather than to develop a shortened psychometric instrument.

The clinical-priority education-gap analysis was exploratory, post hoc and data-driven. The 10 lowest-scoring items were selected pragmatically to summarise the highest-frequency incorrect responses, reviewed for clinical relevance and mapped to practical premature infant care tasks routinely taught by neonatal teams, including feeding tolerance, NPO management, NGT feeding, phototherapy safety, safe handling and umbilical cord care. Incorrect responses across these 10 items were counted for each mother. Five or more gaps indicated incorrect responses on at least half of the selected items and was reported descriptively, not as a validated clinical threshold, formal classification, guideline-derived measure or psychometric scale.

Data were analysed using Stata/SE version 19.0. Categorical variables were summarised as frequencies and percentages, and continuous variables as means and standard deviations, with medians and interquartile ranges where appropriate. Pearson correlations examined relationships between domains. Domain scores were compared using the Friedman test followed by Bonferroni-adjusted Wilcoxon signed-rank tests. Bivariate associations with knowledge category were assessed using Pearson chi-square tests, with Cramer’s V for association strength and Monte Carlo checks where expected cell counts were small.

Ordinal logistic regression was used because the primary outcome was an ordered knowledge category. To reduce overfitting, the primary model included four a priori predictors selected on clinical and conceptual grounds: maternal education, previous premature infants, number of antenatal care (ANC) visits, and specialist premature-care ANC follow-up. Variables were not selected by automated stepwise procedures or solely by bivariate statistical significance. With 170 participants and 41 mothers in the smallest outcome category, the model included 42.5 participants per predictor and 10.3 observations in the smallest category per predictor. The Brant test assessed the proportional odds assumption, and variance inflation factors assessed multicollinearity. Category collapsing was considered but not adopted because the outcome was ordered and the smallest category remained adequate.

Primary planned analyses included descriptive summaries, domain-score comparisons, bivariate associations and the parsimonious adjusted ordinal model. Sensitivity analyses included binary logistic regression comparing poor versus good/excellent knowledge, continuous-score regression, an expanded seven-predictor ordinal model additionally including birth order, prenatal doctor examination and receipt of information after delivery, Monte Carlo checks and hospital fixed-effect models. Potential site-level clustering was examined using hospital indicators because only four hospitals were included, making cluster-robust standard errors, random-effects multilevel modelling and formal intra-class correlation estimation unsuitable as primary approaches. Exploratory analyses included the clinical-priority gap count, domain-specific poor feeding and phototherapy models, adjusted predicted probabilities and the previous premature birth profile. Apart from Bonferroni-adjusted pairwise domain comparisons, no further formal multiplicity correction was applied; exploratory and sensitivity findings were interpreted cautiously using effect sizes, confidence intervals and clinical plausibility.

### Ethical considerations

Ethical approval was granted by the Helsinki Committee (HC) for Ethical Approval, Palestinian Health Research Council (PHRC), Gaza Strip, Palestine (approval number PHRC/HC/1025/22; 07 Feb 2022) in accordance with the Declaration of Helsinki.

### Large language model assistance

ChatGPT was used to support manuscript language editing. The authors verified the final content and take full responsibility for the work.

## Results

### Participant characteristics

Of 172 questionnaires distributed, 170 were completed, giving a response rate of 98.8%. Because demographic or clinical data were not collected from the two mothers who declined participation or did not complete the questionnaire, we could not assess whether they differed from participants. The mean maternal age was 28.8 years (SD 6.4), and 51.2% of mothers had a bachelor’s degree or higher (Table 1). Most mothers were housewives (74.7%), and 77.6% of families had a monthly income below 1973 New Israeli Shekels (Additional file 1, Table S2). Pregnancy-induced hypertension was reported by 45.3% of mothers, previous Caesarean section by 62.4%, and twin pregnancy by 14.1%. Caesarean section was the mode of delivery in 77.1% of index births, and mean gestational age was 34.71 weeks (SD 1.50) (Table 1; Additional file 1, Table S3).

**Table 1:** Maternal sociodemographic, obstetric and infant/birth characteristics of participants

Characteristic	Category	*N* (%)	Mean ± SD
Maternal sociodemographic characteristics			
Maternal age (years)	< 25	50 (29.4)	28.8 ± 6.4
	25–30	58 (34.1)	
	> 30	62 (36.5)	
Maternal education	Primary	4 (2.4)	
	Preparatory	27 (15.9)	
	Secondary	52 (30.6)	
	Bachelor’s degree or higher	87 (51.2)	
Family monthly income (NIS)	< 1973	132 (77.6)	1669 ± 1595
	1973–2470	4 (2.4)	
	> 2470	34 (20.0)	
Maternal obstetric characteristics			
Pregnancy-induced hypertension	Yes	77 (45.3)	
Previous Caesarean section	Yes	106 (62.4)	
Twin pregnancy	Yes	24 (14.1)	
Infant/index-birth characteristics			
Mode of delivery (index birth)	Caesarean section	131 (77.1)	
	Normal vaginal delivery	39 (22.9)	
Gestational age (weeks)	< 34	22 (12.9)	34.71 ± 1.50
	34–35	97 (57.1)	
	> 35	51 (30.0)	

Values are descriptive only. The proportion with pregnancy-induced hypertension reflects this selected hospital-based cohort and sould not be interpreted as population prevalence

*NIS* New Israeli Shekel

### Maternal health services and sources of information

Mothers had a mean of 4.99 antenatal visits (SD 3.69), but only 28.2% reported specialist antenatal follow-up related to premature infant care, defined as targeted antenatal follow-up or counselling concerning premature infant care beyond routine ANC. A prenatal examination by a doctor was reported by 61.2%, and 30.8% of mothers with available data had been told how to prepare for preterm delivery (Additional file 1, Table S4). Only 30.0% received information about premature infant care during pregnancy, whereas 82.4% received information after delivery, mainly from nurses or midwives (Additional file 1, Table S5).

### Maternal knowledge scores and clinical-priority education gaps

The mean overall 30-item knowledge score was 64.1% (SD 22.3; range 13.3–100.0), with knowledge classified as poor in 53 mothers (31.2%), good in 41 (24.1%) and excellent in 76 (44.7%) (Table 2). Infection and skin care had the highest observed percentage score (73.8%), followed by thermoregulation (67.4%), phototherapy (60.6%) and feeding (53.6%). The overall 30-item scale showed high internal consistency (Cronbach’s α = 0.901). However, because the analyses were domain-specific, the domain-level values are more relevant for interpretation: thermoregulation α = 0.773, feeding α = 0.730, phototherapy α = 0.748, and infection and skin care α = 0.593 (Table 2; Additional file 1, Table S6). The infection and skin care domain had the lowest internal consistency, with a mean inter-item correlation of 0.175. Item diagnostics showed that deleting individual items did not materially improve alpha, with the highest alpha-if-item-deleted value being 0.597 (Additional file 1, Table S7).

**Table 2:** Domain-specific and overall maternal knowledge scores

Knowledge domain	Items	Mean ± SD (%)	Range (%)	Median (IQR)	Cronbach’s α
Thermoregulation	10	67.4 ± 24.4	0.0–100.0	70.0 (50.0–90.0)	0.773
Feeding	8	53.6 ± 28.6	0.0–100.0	50.0 (25.0–75.0)	0.730
Phototherapy	5	60.6 ± 32.7	0.0–100.0	70.0 (20.0–80.0)	0.748
Infection and skin care	7	73.8 ± 20.7	0.0–100.0	71.4 (57.1–100.0)	0.593
Overall knowledge	30	64.1 ± 22.3	13.3–100.0	65.0 (43.3–83.3)	0.901

Note. Scores are percentages of correct responses. Overall knowledge was categorised as poor (< 50%), good (50–70%, inclusive) or excellent (> 70%), following Al-Mukhtar and Abdulghani [15], as pragmatic descriptive thresholds rather than validated clinical cut-offs. Overall knowledge was poor in 53 mothers (31.2%), good in 41 (24.1%) and excellent in 76 (44.7%). Domain-level alpha values should be considered for domain-specific interpretation; infection and skin care had lower internal consistency and should be interpreted alongside item-level findings

*IQR* Interquartile range

Knowledge differed significantly across domains (Friedman χ²=104.11, df = 3, *p* < 0.001). Pairwise Wilcoxon signed-rank tests with Bonferroni correction showed that feeding scores were significantly lower than thermoregulation, phototherapy, and infection and skin care, while infection and skin care scores were higher than the other domains (Additional file 1, Table S8). Item-level results by domain are shown in Additional file 1, Tables S9–S12, and the derivation of the 10 lowest-scoring clinical-priority gap items is shown in Additional file 1, Table S13. Across the 10 lowest-scoring items, mothers had a mean of 5.6 clinical-priority education gaps (SD 2.7). As a descriptive summary, 110 mothers (64.7%) had five or more gaps, indicating incorrect responses on at least half of these 10 selected items (Table 3). This threshold was not treated as a validated clinical cut-off.

**Table 3:** Exploratory clinical-priority education gaps based on the 10 lowest-scoring knowledge items

Rank	Education-priority area	Knowledge item	Correct *N* (%)	Incorrect/gap *N* (%)	Practical implication
1	Thermoregulation	Premature babies should not be manipulated frequently	48 (28.2)	122 (71.8)	Reinforce safe handling and heat-loss prevention
2	Feeding	Not all premature babies can tolerate breastfeeding	58 (34.1)	112 (65.9)	Explain feeding readiness and clinical variation
3	Phototherapy	The baby’s genitalia should be covered during phototherapy	70 (41.2)	100 (58.8)	Include phototherapy safety in parent teaching
4	Infection and skin care	The diaper should not cover the umbilical cord	70 (41.2)	100 (58.8)	Reinforce cord care and infection prevention
5	Feeding	Burping a premature baby during and after feeding is essential	73 (42.9)	97 (57.1)	Include practical feeding support before discharge
6	Feeding	Feeding through NGT should not be done by pushing milk through a syringe	83 (48.8)	87 (51.2)	Clarify safe NGT feeding principles
7	Feeding	Pacifiers may be used for NPO premature babies	84 (49.4)	86 (50.6)	Explain non-nutritive sucking and NPO care
8	Phototherapy	The baby’s position should be changed frequently during phototherapy	85 (50.0)	85 (50.0)	Reinforce positioning during phototherapy
9	Infection and skin care	The umbilical cord should not be covered	86 (50.6)	84 (49.4)	Clarify cord exposure and hygiene
10	Feeding	A premature baby may be kept NPO	89 (52.4)	81 (47.6)	Explain why oral feeding may be temporarily withheld

Items are ordered from lowest to highest correct-response proportion. They were selected post hoc as the 10 highest-frequency incorrect responses to provide an exploratory, practice-facing summary. Incorrect responses across these items were counted as the clinical-priority education-gap count; five or more gaps indicated incorrect responses on at least half of the selected items and was descriptive, not a validated clinical cut-off, guideline-derived measure or psychometric scale

*NGT* Nasogastric tube, *NPO* Nil per os

### Associations with maternal knowledge

#### Bivariate associations

In bivariate analysis, knowledge category was associated with previous premature infants (χ²=10.75, Cramer’s V = 0.178, *p* = 0.029), birth order (χ²=14.68, Cramer’s V = 0.208, *p* = 0.023) and number of antenatal visits (χ²=10.66, Cramer’s V = 0.177, *p* = 0.031). Specialist premature-care antenatal follow-up showed a borderline association with knowledge category (χ²=5.85, Cramer’s V = 0.185, *p* = 0.054) (Table 4). Sociodemographic variables, including maternal age, place of residence, maternal education, husband’s education, family income, maternal work status and husband’s work status, were not significantly associated with knowledge category in bivariate analyses. Monte Carlo chi-square sensitivity checks were consistent with the Pearson chi-square results (Additional file 1, Table S14). However, these bivariate findings were considered exploratory and were not interpreted as clinically meaningful on statistical significance alone. Some associations, particularly those involving birth order and ANC visit category, may reflect underlying obstetric history, service-use patterns or residual confounding rather than direct clinical effects.

**Table 4:** Bivariate associations between maternal knowledge category and selected variables

Variable	Category	*N*	Poor *N* (%)	Good *N* (%)	Excellent *N* (%)	χ²	Cramer’s V	*p*-value
Previous premature babies	None	103	32 (31.1)	17 (16.5)	54 (52.4)	10.75	0.178	0.029*
	One child	53	16 (30.2)	18 (34.0)	19 (35.8)			
	Two or more	14	5 (35.7)	6 (42.9)	3 (21.4)			
Number of children, including this baby	< 2 children	46	17 (37.0)	7 (15.2)	22 (47.8)	9.25	0.165	0.055
	2–4 children	91	26 (28.6)	30 (33.0)	35 (38.5)			
	> 4 children	33	10 (30.3)	4 (12.1)	19 (57.6)			
Birth order of index child	First	42	15 (35.7)	5 (11.9)	22 (52.4)	14.68	0.208	0.023*
	Second	38	13 (34.2)	15 (39.5)	10 (26.3)			
	Third	41	8 (19.5)	13 (31.7)	20 (48.8)			
	Fourth or more	49	17 (34.7)	8 (16.3)	24 (49.0)			
ANC visits	< 3 visits	52	16 (30.8)	5 (9.6)	31 (59.6)	10.66	0.177	0.031*
	3–6 visits	70	23 (32.9)	20 (28.6)	27 (38.6)			
	> 6 visits	48	14 (29.2)	16 (33.3)	18 (37.5)			
Specialist premature-care ANC follow-up	Yes	48	10 (20.8)	17 (35.4)	21 (43.8)	5.85	0.185	0.054
	No	122	43 (35.2)	24 (19.7)	55 (45.1)			
Prenatal examination by doctor	Yes	104	33 (31.7)	31 (29.8)	40 (38.5)	5.96	0.187	0.051
	No	66	20 (30.3)	10 (15.2)	36 (54.5)			
Perceived information as adequate†	Yes	57	13 (22.8)	20 (35.1)	24 (42.1)	5.61	0.200	0.060
	No	84	31 (36.9)	16 (19.0)	37 (44.0)			

Percentages are row percentages*P*-values are from Pearson chi-square tests; Cramer’s V indicates association strength. Monte Carlo chi-square sensitivity checks are provided in Additional file 1, Table S14. ANC, antenatal care

*p < 0.05

†*N* = 141 for perceived information as adequate

#### Primary adjusted ordinal regression model

In the parsimonious adjusted ordinal logistic regression model, not receiving specialist premature-care antenatal follow-up was associated with lower odds of higher knowledge category (adjusted OR 0.44, 95% CI 0.20–0.97, *p* = 0.042). A higher category of previous premature infants was also associated with lower odds of higher knowledge (adjusted OR 0.57, 95% CI 0.34–0.96, *p* = 0.033). Maternal education level and number of ANC visits were not significantly associated with knowledge category in the adjusted model (Table 5). The proportional odds assumption was not violated for the primary ordinal model using the Brant test of the parallel regression assumption (global χ²=5.50, df = 4, *p* = 0.240). Multicollinearity diagnostics did not indicate problematic collinearity among predictors in the primary model; variance inflation factor (VIF) values ranged from 1.26 to 1.61.

**Table 5:** Parsimonious adjusted ordinal logistic regression for predictors of higher maternal knowledge category (*N* = 170)

Predictor	Adjusted OR	95% CI	*p*-value
Maternal education level, higher category	0.97	0.66–1.44	0.878
Previous premature infants, higher category	0.57	0.34–0.96	0.033*
Number of ANC visits	0.95	0.87–1.04	0.257
No specialist premature-care ANC follow-up, vs. yes	0.44	0.20–0.97	0.042*

Outcome categories were ordered as poor, good and excellent knowledge. The model was restricted to four a priori predictors to reduce overfitting. OR > 1 indicates higher odds of being in a higher knowledge category; for ordinal predictors, the OR represents a one-category increase. The proportional odds assumption was not violated (Brant global χ²=5.50, df = 4, *p* = 0.240). Nagelkerke pseudo-R²=0.052. VIF values ranged from 1.26 to 1.61

*ANC* Antenatal care, *CI* Confidence interval, *OR* Odds ratio, *VIF* Variance inflation factor

*p < 0.05

#### Sensitivity analyses

Sensitivity analyses supported the main service-related finding. Binary logistic regression showed that absence of specialist follow-up was associated with higher odds of poor knowledge (adjusted OR 3.07, 95% CI 1.11–8.46, *p* = 0.030) (Additional file 1, Table S15). The expanded seven-predictor ordinal model showed a similar direction of association for specialist premature-care ANC follow-up and previous premature infants (Additional file 1, Table S16). Hospital fixed-effect sensitivity analyses did not materially change the findings. Mean overall knowledge scores were similar across hospitals: 64.6% in Al-Shifa, 64.4% in Nasser, 60.9% in Emirati and 62.5% in Al-Aqsa. In the hospital-adjusted ordinal model, absence of specialist premature-care ANC follow-up remained associated with lower odds of higher knowledge category (adjusted OR 0.43, 95% CI 0.19–0.97, *p* = 0.042), and previous premature infants remained associated with lower odds of higher knowledge (adjusted OR 0.56, 95% CI 0.33–0.95, *p* = 0.030). Hospital indicators were not independently associated with knowledge category and did not improve model fit (likelihood-ratio χ²=0.23, df = 3, *p* = 0.973). The clinical-priority education-gap count model was also repeated with hospital indicators, with no material change in the main service-related association (Additional file 1, Table S17).

#### Exploratory analyses

Knowledge domains were positively correlated with each other (all *p* < 0.001; Additional file 1, Table S18). Adjusted predicted probabilities were used as a post-estimation interpretive analysis of the parsimonious ordinal model. Mothers without specialist premature-care antenatal follow-up had a higher predicted probability of poor knowledge than those with follow-up (35.9% vs. 20.3%).

To reduce reliance on the pragmatic knowledge categories, the continuous overall knowledge percentage score was analysed as a sensitivity analysis. A higher category of previous premature infants was associated with an 8.0%-point lower overall knowledge score (95% CI − 15.0 to − 1.1, *p* = 0.024), and absence of specialist premature-care antenatal follow-up was associated with an 11.5%-point lower score (95% CI − 21.6 to − 1.4, *p* = 0.025).

Exploratory models showed that absence of specialist premature-care antenatal follow-up was associated with poor phototherapy knowledge (adjusted OR 2.95, 95% CI 1.14–7.59, *p* = 0.025) and more clinical-priority education gaps (IRR 1.28, 95% CI 1.04–1.57, *p* = 0.019), while a higher category of previous premature infants was also associated with more clinical-priority education gaps (IRR 1.21, 95% CI 1.06–1.37, *p* = 0.004). No prenatal doctor examination was associated with fewer clinical-priority education gaps, but this borderline finding was clinically difficult to interpret and was not used to support clinical interpretation (IRR 0.82, 95% CI 0.67–1.00, *p* = 0.049). Full exploratory and sensitivity analyses are shown in Additional file 1, Table S19.

#### Descriptive profile by previous premature birth history

As an exploratory descriptive analysis, we examined whether the association between previous premature infants and lower knowledge category reflected broader demographic or service-use patterns. The profile is shown in Additional file 1, Table S20. Mothers with previous premature infants were older, had more children, reported more ANC visits, and were more likely to report specialist premature-care ANC follow-up and premature-care information during pregnancy. Despite this greater reported service exposure, excellent knowledge decreased with previous premature birth history: 52.4% among mothers with no previous premature infant, 35.8% among those with one, and 21.4% among those with two or more.

## Discussion

Previous studies have reported variable maternal knowledge or awareness of premature infant care. Poor knowledge was reported in 53.0% of mothers in Mosul, Iraq [15], a Rwandan study reported a mean awareness score of 59.3% [16], Aldirawi et al. reported an overall post-discharge maternal knowledge value of 58.4% in Gaza [17], and Abdullah and Hassan reported poor awareness of preterm baby home care in Erbil, Iraq [18]. These comparisons should be interpreted cautiously because studies differed in sampling, questionnaire design, timing and scoring. The main contribution of the present study is therefore not the moderate overall score alone, but the finding that this score masked task-specific gaps in clinically important areas. This matters because neonatal parent education is usually delivered through practical conversations about feeding, temperature control, phototherapy and infection prevention, rather than through general knowledge alone.

The domain-specific findings reinforce this point. Infection and skin care had the highest observed percentage score, consistent with previous Gaza-based findings [17], but its relatively low internal consistency (α = 0.593) means that it should be interpreted cautiously and alongside item-level findings rather than as a strongly reliable domain score. Important gaps remained in hand hygiene and umbilical cord care, which are central to neonatal infection prevention and can be taught, checked and reinforced before discharge [19, 20]. Feeding was the weakest domain, consistent with studies from Rwanda and Brazil showing that feeding and home-care knowledge can be challenging for mothers of premature infants [16, 21]. This is clinically important because premature infant feeding often involves staged decisions about feeding readiness, temporary NPO management, NGT feeding, feeding advancement and transition to oral feeding, with decisions shaped by infant maturity, respiratory stability, sucking and swallowing coordination, feeding tolerance and overall condition [22, 23]. Mothers’ understanding and confidence around feeding also develop through early feeding experience [24], while broader nutritional requirements and post-discharge micronutrient supplementation are important in preterm infant care [25, 26]. Thermoregulation gaps, particularly around frequent handling and heat-loss prevention, are also important given known challenges in maternal knowledge of thermal care and the clinical importance of maintaining appropriate body temperature in very-low-birth-weight infants [27–29]. Phototherapy findings similarly suggested gaps around positioning, protection and monitoring. Previous studies show that maternal and caregiver knowledge of neonatal jaundice and its management varies [30, 31], while clinical guidance highlights the importance of appropriate monitoring and protection during phototherapy [32]. 

The exploratory clinical-priority education-gap analysis was one of the most practice-facing elements of the study, but it should be interpreted as a pragmatic summary rather than a validated scale. The 10 items were selected post hoc because they had the highest proportions of incorrect responses and mapped to modifiable care tasks that neonatal teams routinely teach or supervise, including feeding tolerance, NPO management, NGT feeding, phototherapy safety, handling practices and umbilical cord care. In resource-constrained neonatal settings, a short checklist based on these topics could help standardise minimum education content during antenatal counselling, bedside teaching and discharge preparation. However, such a checklist should be considered an illustrative framework derived from the present findings, not a validated intervention or guideline-derived measure.

The most actionable service-related finding was the association between specialist premature-care antenatal follow-up and higher maternal knowledge, which remained evident in the parsimonious adjusted model and after hospital fixed-effect sensitivity analysis. However, the model had low explanatory power, so these associations should be interpreted as identifying potentially relevant service-related and experiential factors rather than as a comprehensive prediction model. Antenatal care has been linked with newborn-care knowledge in other settings [33], and WHO guidance emphasises repeated antenatal contacts beginning early in pregnancy [34]. In this study, only 28.2% of mothers reported specialist antenatal follow-up related to premature infant care, despite all participants ultimately delivering prematurely. This suggests that the issue is not simply the number of antenatal contacts, but whether they include specific preparation for premature infant care, including feeding readiness, temporary NPO management, NGT feeding, thermal protection, phototherapy safety and infection prevention. Broader socioeconomic, psychosocial and food-security factors associated with preterm labour also emphasise the need to interpret antenatal education within the wider conditions in which mothers receive care [35]. Structured antenatal education can improve newborn-care knowledge [36], and in Gaza, where services operate under sustained pressure, strengthening the educational content of existing antenatal contacts may be more feasible than relying only on additional visits [9, 37]. 

Not all statistically significant or borderline findings were equally actionable. Specialist premature-care antenatal follow-up was the most clinically interpretable association because it was consistent across analyses and had a plausible service-delivery explanation. By contrast, bivariate associations with birth order and antenatal care visit category, and the exploratory association between no prenatal doctor examination and fewer clinical-priority education gaps, are more difficult to interpret clinically and may reflect obstetric history, healthcare-seeking patterns, case complexity or residual confounding. They should therefore be viewed as exploratory or hypothesis-generating rather than immediate targets for practice change.

The humanitarian and resource-constrained context of Gaza is central to interpreting our findings. Maternal education in neonatal care is shaped by disrupted services, overcrowded hospitals, staffing pressure and limited time for counselling. Mothers may also receive information during periods of anxiety, fatigue or uncertainty, which may affect understanding and retention. The persistence of clinically important gaps despite high postnatal information exposure suggests that the issue may not be information exposure alone, but whether education is structured, repeated, practical and checked for understanding. In this context, a domain-specific education framework may help standardise essential counselling content without replacing clinical judgement or individualised advice.

Postnatal education remains important, but receipt of information after delivery was not independently associated with higher knowledge category. This should not be interpreted as evidence that postnatal education is ineffective; rather, the content, timing, quality and retention of information may have varied. Previous work from Gaza and Brazil has highlighted the role of neonatal nurses in educating mothers of premature infants [17, 21], and prematurity-related knowledge has also been examined among parents of very preterm infants [38]. Evidence on parent-provider communication during neonatal hospitalisation suggests that information needs to be structured, consistent and responsive to parents’ needs [39]. Few mothers reported mass media as an information source during pregnancy. Parent-targeted postnatal education interventions in low- and middle-income countries may raise awareness [40], but they cannot replace structured, clinically grounded education within the care pathway.

Maternal education and family income were not significantly associated with knowledge category, contrasting with studies from Iraq, India, Nepal and Ethiopia where maternal education was associated with knowledge of premature infant care, home-based neonatal care, newborn care or neonatal danger signs [15, 41–43]. This may reflect limited variation in formal education within the sample, or the possibility that access to targeted clinical information is more influential than general educational background in highly constrained settings. This challenges the assumption that formally educated mothers necessarily have adequate knowledge of premature infant care.

Previous premature birth was associated with lower odds of higher knowledge and with more priority gaps, which is counterintuitive and should be interpreted cautiously as an association, not a causal finding. In this cohort, mothers with previous premature infants were older, had more children, reported more ANC visits, and were more likely to report specialist premature-care ANC follow-up and premature-care information during pregnancy, suggesting that the association did not simply reflect lower service contact. Possible explanations include more complex reproductive histories, repeated stressful neonatal experiences, variable counselling quality, or residual confounding by unmeasured factors such as severity of previous neonatal illness, maternal psychological distress, family support, health literacy or post-discharge follow-up. The practical implication is that previous experience of prematurity should not be assumed to provide adequate preparation; these mothers may still need structured, individualised education with explicit checking of understanding.

The exploratory association between no prenatal doctor examination and fewer clinical-priority education gaps was also clinically difficult to interpret and should not be taken to suggest that absence of doctor examination is beneficial. It may reflect residual confounding, case complexity or measurement limitations, because the questionnaire recorded whether prenatal doctor examination occurred but not the quality, content, timing or educational component of that contact. This finding should therefore remain hypothesis-generating and should not guide practice without further study.

This study has several strengths. It was conducted across multiple government hospitals, achieved a high response rate and examined knowledge both as an overall score and across clinically meaningful domains. The item-level analysis also improved clinical interpretability by identifying the specific areas where education should be prioritised. The statistical approach was strengthened by Monte Carlo sensitivity checks, assessment of the proportional odds assumption and exploratory analyses using alternative outcomes. These analyses supported the main finding that specialist premature-care antenatal follow-up was associated with better maternal knowledge.

This study has several limitations. Its cross-sectional design prevents causal inference. The questionnaire was researcher-designed and not directly adapted from a validated instrument, which may limit comparability with other studies. Although it was developed from the literature, translated into Arabic, reviewed by seven specialists and piloted before data collection, formal back-translation records and quantitative content-validity indices were not available for reporting. The poor, good and excellent categories were pragmatic descriptive thresholds rather than clinically validated cut-offs and should be interpreted alongside the continuous-score sensitivity analysis. The infection and skin care domain also had lower internal consistency, so findings from this domain should be interpreted cautiously, with emphasis on item-level results. Consecutive non-random sampling was a pragmatic approach during neonatal admission and may have affected representativeness. Non-participant characteristics were not available for comparison, and findings may not generalise to all mothers of premature infants in Gaza, private hospitals, non-hospital settings or culturally different populations. Self-reported and interviewer-administered responses may have been affected by recall, social desirability and interviewer bias. Several service-related variables were mother-reported yes/no items, so the questionnaire did not capture the content, quality, timing, frequency or duration of these contacts, and interview timing during admission was not standardised. More broadly, maternal experiences of caring for preterm infants are shaped by social vulnerability, family circumstances and health-system pressures [44]. The data were also collected before the major escalation in Gaza from October 2023 and therefore describe maternal knowledge before the most recent deterioration in the healthcare context. Although the primary model was restricted to four a priori predictors, the sample size limited precision, the model had low explanatory power, and sensitivity and exploratory analyses should be interpreted as robustness checks or hypothesis-generating analyses. Finally, the clinical-priority education-gap count was derived post hoc, hospital fixed-effect analyses were limited by the small number of hospitals, detailed hospital-level practice characteristics were not measured, and unmeasured factors such as psychological stress, health literacy, previous counselling quality, previous neonatal illness severity and post-discharge support may have influenced some findings.

These findings can also be interpreted in relation to WHO guidance on care of preterm or low-birth-weight infants, which emphasises family involvement, parental support and education as part of routine care [13]. In this context, the identified knowledge gaps may represent barriers to meaningful maternal participation in feeding support, thermal care, infection prevention, phototherapy-related care and discharge preparation. Future studies should test whether structured antenatal and postnatal education tools, including “explain, demonstrate and check understanding” approaches, improve maternal knowledge, caregiving confidence, caregiving practices and infant outcomes, and whether remote support, such as video consultation, can support early in-home care after discharge [45]. 

## Conclusions

This study found moderate overall maternal knowledge of premature infant care in Gaza, but this masked important gaps in feeding, phototherapy safety, handling practices and umbilical cord care. Specialist premature-care antenatal follow-up was associated with better knowledge and fewer clinical-priority education gaps, although the cross-sectional design precludes causal inference. Previous premature birth was associated with lower knowledge, indicating that prior experience should not be assumed to provide adequate preparation. These findings highlight specialist premature-care antenatal follow-up as a potential opportunity for structured, domain-specific parent education in resource-constrained neonatal settings; future studies should evaluate whether such tools improve maternal knowledge, caregiving confidence, caregiving practices and infant outcomes.

## Supplementary Information

Supplementary Material 1.

## Data Availability

The datasets used and analysed during the current study are not publicly available because the ethical approval and consent procedures did not include unrestricted public data sharing, and because of participant privacy considerations. De-identified data are available from the corresponding author on reasonable request, subject to ethical approval and data protection regulations.

## Abbreviations

ANC: Antenatal care
NGT: Nasogastric tube
NIS: New Israeli Shekel
NPO: Nil per os
CI: Confidence interval
IRR: Incidence rate ratio
OR: Odds ratio
SD: Standard deviation
WHO: World Health Organization
