# Supplementary material for: Maternal knowledge and clinically relevant care gaps in preterm infant care in Gaza, Palestine: a cross-sectional study
Source: BMC Pediatr. 2026 Jun 23;26:833. doi: 10.1186/s12887-026-07188-5 (PMC13548630; doi:10.1186/s12887-026-07188-5)
Supplement: Supplementary file 1 [file 12887_2026_7188_MOESM1_ESM.pdf]

Additional file 1. Supplementary materials

Article title: Maternal Knowledge and Clinically Relevant Care Gaps in Preterm Infant Care in Gaza, Palestine: A Cross-Sectional Study

Journal: BMC Pediatrics

Authors: Mohammed Abdeljawad, Ahmed Najim, Hamza Abdeljawad, Josie Rodgers, Reham Almukbel, Kinan Mokbel

Corresponding author: Prof Kinan Mokbel
Department of Health and Care Professions, College of Medicine and Health, University of Exeter Medical School, Exeter, UK
Email: K.A.Mokbel@exeter.ac.uk

This supplementary file contains Supplementary Tables S1–S20 and the English and Arabic questionnaires.

Appendix 1. Supplementary tables

Supplementary Table S1. Proportionate sample size by hospital

Hospital	Estimated admissions/year	Estimated admissions/2 months	Required sample
Al-Shifa Medical Complex	996	166	100
Nasser Hospital	437	72	43
Emirati Hospital	147	24	15
Al-Aqsa Hospital	128	21	14
Total	1708	283	172

Note. *These figures were based on hospital admission estimates and were used only for proportional sample allocation; they were not participant-level data.*

Supplementary Table S2. Full sociodemographic characteristics of participants (N=170)

Characteristic	Category	N (%)	Mean ± SD
Maternal age (years)	<25	50 (29.4)	28.8 ± 6.4
	25–30	58 (34.1)	
	>30	62 (36.5)	
Place of residence	Rafah	23 (13.5)	
	Khan Younes	38 (22.4)	
	Middle Gaza	20 (11.8)	
	Gaza	66 (38.8)	
	North Gaza	23 (13.5)	
Maternal education	Primary	4 (2.4)	
	Preparatory	27 (15.9)	
	Secondary	52 (30.6)	
	Bachelor’s degree or more	87 (51.2)	
Husband’s education	Primary	4 (2.4)	
	Preparatory	12 (7.1)	
	Secondary	52 (30.6)	
	Bachelor’s degree or more	102 (60.0)	

Family monthly income (NIS)	<1973	132 (77.6)	1669 ± 1595
	1973–2470	4 (2.4)	
	>2470	34 (20.0)	
Mother’s work status	Employed/worker	43 (25.3)	
	Housewife	127 (74.7)	
Husband’s work status	Employed/worker	133 (78.2)	
	Not working	37 (21.8)	

Note. *The term ‘husband’ reflects the wording used in the locally reviewed questionnaire and was considered contextually appropriate by the expert panel during questionnaire development.*

Supplementary Table S3. Obstetric characteristics of participants (N=170)

Characteristic	Category	N (%)	Mean ± SD
Diabetes mellitus	Yes	10 (5.9)	
	No	160 (94.1)	
Pregnancy-induced hypertension	Yes	77 (45.3)	
	No	93 (54.7)	
Premature rupture of membranes	Yes	8 (4.7)	
	No	162 (95.3)	
Previous Caesarean section	Yes	106 (62.4)	
	No	64 (37.6)	
Twin pregnancy	Yes	24 (14.1)	
	No	146 (85.9)	
Mode of delivery	Normal vaginal delivery	39 (22.9)	
	Caesarean section	131 (77.1)	
Previous premature babies	None	103 (60.6)	
	One child	53 (31.2)	
	Two or more	14 (8.2)	
Gestational age (weeks)	<34	22 (12.9)	34.71 ± 1.50
	34–35	97 (57.1)	
	>35	51 (30.0)	
Number of children including this baby	<2 children	46 (27.1)	2.81 ± 1.74
	2–4 children	91 (53.5)	
	>4 children	33 (19.4)	
Birth order	First	42 (24.7)	
	Second	38 (22.4)	
	Third	41 (24.1)	
	Fourth or more	49 (28.8)	
Sex of baby	Male	82 (48.2)	
	Female	88 (51.8)	

Supplementary Table S4. Maternal health services received (N=170 unless otherwise stated)

Variable	Category	N (%)	Mean ± SD
Number of ANC visits	<3 visits	52 (30.6)	4.99 ± 3.69
	3–6 visits	70 (41.2)	
	>6 visits	48 (28.2)	
Specialist premature-care ANC follow-up	Yes	48 (28.2)	
	No	122 (71.8)	
Prenatal examination by doctor	Yes	104 (61.2)	
	No	66 (38.8)	
Prenatal examination by nurse/midwife	Yes	99 (58.2)	
	No	71 (41.8)	
Told how to prepare for preterm delivery*	Yes	52 (30.8)	

	No	117 (69.2)	

***N=169** *for "told how to prepare for preterm delivery" because one response was missing.*

Supplementary Table S5. Sources of information about premature infant care

Variable	Category	N (%)	Denominator
Received information during pregnancy	Yes	51 (30.0)	170
	No	119 (70.0)	170
Doctor, during pregnancy	Yes	43 (84.3)	51
Nurse/midwife, during pregnancy	Yes	14 (27.5)	51
Mass media, during pregnancy	Yes	3 (5.9)	51
Relative/friend, during pregnancy	Yes	5 (9.8)	51
Received information after delivery	Yes	140 (82.4)	170
	No	30 (17.6)	170
Doctor, after delivery	Yes	55 (39.3)	140
Nurse/midwife, after delivery	Yes	124 (88.6)	140
Mass media, after delivery	Yes	8 (5.7)	140
Relative/friend, after delivery	Yes	22 (15.7)	140
Perceived information as adequate	Yes	57 (40.4)	141
	No	84 (59.6)	141

Supplementary Table S6. Reliability summary and interpretation of the knowledge scale

Scale/domain	Number of items	Cronbach's α	Interpretation for this study
Thermoregulation	10	0.773	Supported domain-level interpretation alongside item-level findings.
Feeding	8	0.730	Supported domain-level interpretation alongside item-level findings.
Phototherapy	5	0.748	Supported domain-level interpretation alongside item-level findings.
Infection and skin care	7	0.593	Lower internal consistency; interpreted cautiously and alongside item-level findings and item diagnostics in Supplementary Table S7.
Overall knowledge scale	30	0.901	Supported use of the overall 30-item knowledge score, while domain-level alpha values were considered more relevant for domain-specific interpretation.

Note. *Cronbach's alpha was interpreted as equivalent to Kuder-Richardson 20 because the knowledge items were dichotomously scored. This table provides reliability values separately from the domain scores reported in Table 2. The infection and skin care domain had the lowest internal consistency and was therefore examined further in Supplementary Table S7.*

Supplementary Table S7. Item diagnostics for the infection and skin care domain

Item	Correct N (%)	Corrected item-total correlation	Cronbach's alpha if item deleted
The diaper can cover the umbilical cord	70 (41.2)	0.487	0.476
The umbilical cord should be kept dry and clean	160 (94.1)	0.242	0.579
A soiled diaper should be changed immediately	164 (96.5)	0.187	0.591
Hand hygiene should be done before handling a premature baby	97 (57.1)	0.511	0.463
The umbilical cord should be covered	86 (50.6)	0.241	0.595
Instruments used for the care of the baby could be shared with others	161 (94.7)	0.150	0.597

Perineal and umbilical cord areas should be monitored closely for infection	140 (82.4)	0.371	0.533

Note. *The infection and skin care domain had a Cronbach's alpha of 0.593. Deleting individual items did not materially improve alpha. The lower internal consistency may reflect the heterogeneity of behaviours included in this domain and restricted variance in items with very high correct-response rates.*

Supplementary Table S8. Comparison of knowledge scores across domains

Comparison	Mean difference, percentage points	Median difference	Adjusted *p*-value
Thermoregulation vs feeding	13.8	15.0	<0.001
Thermoregulation vs phototherapy	6.8	10.0	0.002
Thermoregulation vs infection and skin care	−6.4	−5.7	<0.001
Feeding vs phototherapy	−7.0	−5.0	0.005
Feeding vs infection and skin care	−20.2	−21.4	<0.001
Phototherapy vs infection and skin care	−13.2	−17.1	<0.001

Note. *Domain scores were compared using the Friedman test, which showed a statistically significant difference across the four domains (χ^2=104.11, df=3, p<0.001). Pairwise comparisons were conducted using Wilcoxon signed-rank tests with Bonferroni correction for multiple comparisons. Positive mean and median differences indicate higher scores in the first listed domain.*

Supplementary Table S9. Thermoregulation knowledge items (N=170)

Item	Correct N (%)	Incorrect N (%)	Rank
The premature baby should be placed in a warm environment	106 (62.4)	64 (37.6)	5
The premature baby loses body heat rapidly if uncovered	91 (53.5)	79 (46.5)	9
Covering a premature baby's head helps keep the baby's temperature	142 (83.5)	28 (16.5)	4
Premature babies should be placed near open windows with air drafts	153 (90.0)	17 (10.0)	2
A premature baby could be placed on cold beds or blankets	95 (55.9)	75 (44.1)	8
Premature babies should be manipulated frequently	48 (28.2)	122 (71.8)	10
A source of heat is essential to keep a baby's temperature normal	96 (56.5)	74 (43.5)	7
Handling the baby with warmed hands is necessary	145 (85.3)	25 (14.7)	3
Dressing the baby in previously warmed clothes is necessary	106 (62.4)	64 (37.6)	6
The baby should be bathed with warm water and immediately dried and covered	164 (96.5)	6 (3.5)	1

Supplementary Table S10. Feeding knowledge items (N=170)

Item	Correct N (%)	Incorrect N (%)	Rank
Any premature baby can tolerate breastfeeding	58 (34.1)	112 (65.9)	8
A premature baby may be kept NPO	89 (52.4)	81 (47.6)	4
A premature baby may be given feeding through NGT	106 (62.4)	64 (37.6)	3
Feeding a premature baby through NGT is by pushing milk through a syringe in the tube	83 (48.8)	87 (51.2)	6
Pacifiers may be used for NPO premature baby	84 (49.4)	86 (50.6)	5
Premature babies should receive small and frequent amounts of milk	120 (70.6)	50 (29.4)	1
Burping a premature baby during and after feeding is not essential	73 (42.9)	97 (57.1)	7
Expressed breast milk is best if the baby is unable to breastfeed	116 (68.2)	54 (31.8)	2

Supplementary Table S11. Phototherapy knowledge items (N=170)

Item	Correct N (%)	Incorrect N (%)	Rank
The premature baby's temperature should be monitored during phototherapy	148 (87.1)	22 (12.9)	1
The premature baby's position should be changed frequently during phototherapy	85 (50.0)	85 (50.0)	4

The baby's eyes should be covered during phototherapy	94 (55.3)	76 (44.7)	3
The baby's genitalia should be covered during phototherapy	70 (41.2)	100 (58.8)	5
A baby under phototherapy should be kept well hydrated through IV fluid or milk feeding	118 (69.4)	52 (30.6)	2

Supplementary Table S12. Infection and skin care knowledge items (N=170)

Item	Correct N (%)	Incorrect N (%)	Rank
The diaper can cover the umbilical cord	70 (41.2)	100 (58.8)	7
The umbilical cord should be kept dry and clean	160 (94.1)	10 (5.9)	3
A soiled diaper should be changed immediately	164 (96.5)	6 (3.5)	1
Hand hygiene should be done before handling a premature baby	97 (57.1)	73 (42.9)	5
The umbilical cord should be covered	86 (50.6)	84 (49.4)	6
Instruments used for the care of the baby could be shared with others	161 (94.7)	9 (5.3)	2
Perineal and umbilical cord areas should be monitored closely for infection	140 (82.4)	30 (17.6)	4

Supplementary Table S13. Derivation of the clinical-priority education-gap count from the 10 lowest-scoring items

Rank	Questionnaire item	Domain	Correct response/interpretation	Correct N (%)	Incorrect/gap N (%)
1	A6	Thermoregulation	Premature babies should not be manipulated frequently	48 (28.2)	122 (71.8)
2	B1	Feeding	Not all premature babies can tolerate breastfeeding	58 (34.1)	112 (65.9)
3	C4	Phototherapy	The baby's genitalia should be covered during phototherapy	70 (41.2)	100 (58.8)
4	D1	Infection and skin care	The diaper should not cover the umbilical cord	70 (41.2)	100 (58.8)
5	B7	Feeding	Burping a premature baby during and after feeding is essential	73 (42.9)	97 (57.1)
6	B4	Feeding	Feeding through NGT should not be done by pushing milk through a syringe	83 (48.8)	87 (51.2)
7	B5	Feeding	Pacifiers may be used for NPO premature babies	84 (49.4)	86 (50.6)
8	C2	Phototherapy	The baby's position should be changed frequently during phototherapy	85 (50.0)	85 (50.0)
9	D5	Infection and skin care	The umbilical cord should not be covered	86 (50.6)	84 (49.4)
10	B2	Feeding	A premature baby may be kept NPO	89 (52.4)	81 (47.6)

Note. *This table documents the item codes, ranking and scoring basis used to derive the clinical-priority education-gap count. Items are ordered from lowest to highest correct-response proportion and correspond to the 10 lowest-scoring items across the 30-item knowledge section. Incorrect responses across these items were counted as exploratory clinical-priority education gaps. Five or more gaps indicated incorrect responses on at least half of the selected items and was descriptive only, not a validated clinical cut-off, guideline-derived measure or psychometric scale. NGT, nasogastric tube; NPO, nil per os.*

Supplementary Table S14. Monte Carlo sensitivity checks for selected bivariate associations

Variable	Pearson χ^2	Pearson *p*-value	Minimum expected count	Monte Carlo *p*-value	Interpretation
Previous premature babies	10.75	0.029	3.38	**0.029***	Significant
Number of children, including this baby	9.25	0.055	7.96	0.054	Borderline

Birth order of index child	14.68	0.023	9.16	**0.022***	Significant
ANC visits group	10.66	0.031	11.58	**0.030***	Significant
Specialist premature-care ANC follow-up	5.85	0.054	11.58	0.052	Borderline
Prenatal examination by doctor	5.96	0.051	15.92	0.051	Borderline
Perceived information as adequate	5.61	0.060	14.55	0.057	Borderline
Mass media during pregnancy	3.10	0.212	0.82	0.178	Not significant; sparse

Note. *Monte Carlo chi-square sensitivity checks used 200,000 simulated tables. ANC, antenatal care. *p<0.05. Borderline refers to p-values close to, but not below, 0.05.*

Supplementary Table S15. Sensitivity binary logistic regression: poor versus good/excellent knowledge

Predictor	Adjusted OR for poor knowledge	95% CI	*p*-value
Maternal education level, higher category	0.88	0.56–1.39	0.583
Previous premature infants, higher category	1.45	0.77–2.71	0.246
Birth order of index child, higher category	0.90	0.65–1.25	0.534
Number of ANC visits	0.98	0.87–1.10	0.697
No specialist premature-care ANC follow-up, vs yes	3.07	1.11–8.46	**0.030***
No prenatal doctor examination, vs yes	0.52	0.20–1.31	0.162
No information after delivery, vs yes	0.84	0.34–2.10	0.711

Note. *Outcome was poor knowledge versus good/excellent knowledge. This model was used as a sensitivity analysis to the ordinal model. OR >1 indicates higher odds of poor knowledge. ANC, antenatal care; CI, confidence interval; OR, odds ratio. *p<0.05.*

Supplementary Table S16. Expanded seven-predictor ordinal logistic regression sensitivity model

Predictor	Adjusted OR	95% CI	p-value
Maternal education level, higher category	1.07	0.71–1.63	0.732
Previous premature infants, higher category	0.51	0.29–0.88	**0.016***
Birth order of index child, higher category	1.22	0.91–1.64	0.175
Number of ANC visits	0.99	0.89–1.09	0.789
No specialist premature-care ANC follow-up, vs yes	0.34	0.15–0.80	**0.013***
No prenatal doctor examination, vs yes	2.28	0.99–5.26	0.054
No information after delivery, vs yes	1.34	0.58–3.08	0.494

Note. *This expanded model was retained as a sensitivity analysis; the primary model was restricted to four a priori predictors to reduce overfitting. Outcome categories were ordered as poor, good and excellent knowledge. OR >1 indicates higher odds of being in a higher knowledge category. Multicollinearity diagnostics did not indicate problematic collinearity; the highest VIF was 1.97. ANC, antenatal care; CI, confidence interval; OR, odds ratio. *p<0.05.*

Supplementary Table S17. Hospital fixed-effect sensitivity analyses

Predictor	Adjusted estimate	95% CI	*p*-value
Maternal education level, higher category†	OR 0.98	0.65–1.46	0.906
Previous premature infants, higher category†	OR 0.56	0.33–0.95	**0.030***
Number of ANC visits†	OR 0.95	0.86–1.04	0.268
No specialist premature-care ANC follow-up, vs yes†	OR 0.43	0.19–0.97	**0.042***
Nasser, vs Al-Shifa†	OR 0.94	0.47–1.89	0.865
Emirati, vs Al-Shifa†	OR 0.93	0.32–2.70	0.899
Al-Aqsa, vs Al-Shifa†	OR 1.24	0.40–3.88	0.706
No specialist premature-care ANC follow-up, vs yes‡	IRR 1.28	1.04–1.58	**0.019***
Previous premature infants, higher category‡	IRR 1.21	1.06–1.37	**0.004***

Note. *Hospital fixed-effect sensitivity analyses were conducted using Al-Shifa as the reference hospital. †Ordinal logistic regression model including the four a priori predictors plus hospital indicators. ‡Poisson regression model for the clinical-priority gap count, repeated with hospital indicators and robust standard errors. Hospital indicators did not improve ordinal model fit (likelihood-ratio* χ^2*=0.23, df=3, p=0.973). ANC, antenatal care; CI, confidence interval; IRR, incidence rate ratio; OR, odds ratio. *p<0.05.*

Supplementary Table S18. Pearson correlations between knowledge domains (N=170)

Domain comparison	Pearson correlation coefficient (r)	*p*-value
Thermoregulation and feeding	0.735	<0.001
Thermoregulation and phototherapy	0.670	<0.001
Thermoregulation and infection and skin care	0.586	<0.001
Feeding and phototherapy	0.633	<0.001
Feeding and infection and skin care	0.570	<0.001
Phototherapy and infection and skin care	0.574	<0.001

Supplementary Table S19. Additional exploratory and sensitivity analyses

Predictor	Poor feeding OR (95% CI); *p*-value	Poor phototherapy OR (95% CI); *p*-value	Continuous score beta (95% CI); *p*-value	Clinical-priority gap count IRR (95% CI); *p*-value
Maternal education level, higher category	0.75 (0.48–1.18); 0.212	1.05 (0.67–1.63); 0.831	0.2 (−5.0 to 5.3); 0.944	1.01 (0.92–1.13); 0.780
Previous premature infants, higher category	1.49 (0.83–2.70); 0.183	1.50 (0.82–2.72); 0.186	**−8.0 (−15.0 to −1.1); 0.024***	**1.21 (1.06–1.37); 0.004***
Birth order of index child, higher category	0.91 (0.67–1.25); 0.571	1.02 (0.75–1.38); 0.919	2.6 (−0.9 to 6.1); 0.142	0.96 (0.90–1.03); 0.312
Number of ANC visits	1.00 (0.90–1.12); 0.982	0.97 (0.86–1.08); 0.565	−0.1 (−1.3 to 1.1); 0.880	1.00 (0.97–1.03); 0.998
No specialist premature-care ANC follow-up, vs yes	1.38 (0.55–3.48); 0.491	**2.95 (1.14–7.59); 0.025***	**−11.5 (−21.6 to −1.4); 0.025***	**1.28 (1.04–1.57); 0.019***
No prenatal doctor examination, vs yes	0.48 (0.19–1.19); 0.113	0.62 (0.25–1.52); 0.300	8.7 (−1.4 to 18.8); 0.091	**0.82 (0.67–1.00); 0.049***
No information after delivery, vs yes	1.21 (0.51–2.91); 0.663	0.53 (0.22–1.31); 0.171	3.7 (−6.8 to 14.1); 0.492	1.00 (0.82–1.22); 0.968

Note. *Poor feeding and poor phototherapy knowledge were defined as domain scores below 50%. The continuous-score model used the overall 30-item knowledge percentage score with robust standard errors. The clinical-priority gap count was the number of incorrect responses across the 10 lowest-scoring knowledge items and was analysed using Poisson regression with robust standard errors. *p<0.05. ANC, antenatal care; CI, confidence interval; IRR, incidence rate ratio; OR, odds ratio.*

Supplementary Table S20. Exploratory profile of mothers by previous premature birth history

Variable	No previous premature infant	One previous premature infant	Two or more previous premature infants	*p*-value
N	103	53	14	
Overall knowledge score, mean ± SD	66.5 ± 22.4	61.1 ± 22.4	57.6 ± 20.4	0.190
Poor knowledge, N (%)	32 (31.1)	16 (30.2)	5 (35.7)	0.029
Good knowledge, N (%)	17 (16.5)	18 (34.0)	6 (42.9)	0.029
Excellent knowledge, N (%)	54 (52.4)	19 (35.8)	3 (21.4)	0.029
Maternal age, mean ± SD	27.0 ± 6.2	31.8 ± 5.4	30.4 ± 7.2	<0.001
Number of children, mean ± SD	2.35 ± 1.70	3.45 ± 1.58	3.71 ± 1.59	<0.001
ANC visits, mean ± SD	3.87 ± 3.33	6.64 ± 3.74	6.93 ± 3.02	<0.001

Specialist premature-care ANC follow-up, yes, N (%)	11 (10.7)	28 (52.8)	9 (64.3)	<0.001
Received premature-care information during pregnancy, yes, N (%)	13 (12.6)	30 (56.6)	8 (57.1)	<0.001
Told how to prepare for preterm delivery, yes, N (%)	17 (16.5)	27 (50.9)	8 (61.5)	<0.001

Note*. This table explores the association between previous premature birth history and knowledge category. P-values for continuous variables are from Kruskal-Wallis tests, and p-values for categorical variables are from Pearson chi-square tests. The p-value for knowledge category applies to the overall poor, good and excellent distribution. One response was missing for "told how to prepare for preterm delivery" among mothers with two or more previous premature infants. ANC, antenatal care; SD, standard deviation.*

Appendix 2. Study questionnaire

Interviewer-administered questionnaire (English version)

Please answer the following questions

Part I: Socio-demographic characteristics

- Age in years:……..years
- Place of residence: …………………
- Years of education:…………years
- Average family monthly income:……….NIS
- Husband's years of education:……………..years
- Mother's work status: 1- Employed/worker 2- Housewife
- Husband's work status: 1- Employed/worker 2- Not working

Part II: Obstetric factors

1. Maternal risk factors

Diabetes mellitus ☐ Pregnancy-induced hypertension ☐ Premature rupture of ☐ membranes
Previous Caesarean section ☐ Twins pregnancy. ☐
☐ Others (specify……………….)

2. Mode of delivery for this baby:

☐ Normal vaginal delivery ☐ Assisted vaginal delivery (vacuum) ☐ Caesarean section

3. No. of previous premature babies:…………………..
4. Gestational age of this baby in weeks:………………..Weeks
5. No. of children, including this baby:……………………..
6. Birth order of this child: 1- First 2- Second 3- Third 4- Fourth or more
7. Sex of this baby: 1- Male 2- Female
8. The birth date of the baby:……………………………….

Part III: Maternal health services

1- Number of antenatal visits during pregnancy of this baby:……………times

2- Did you receive a special ANC follow-up related to the care of a premature baby? ☐ Yes ☐ No

3- Did you receive a prenatal exam at ANC from a doctor? ☐ Yes ☐ No

4- Did you receive a prenatal exam at ANC from a nurse/midwife? ☐Yes ☐ No

5- Did a healthcare provider tell you how to prepare for preterm delivery?

☐ Yes ☐ No

Part IV: Source of information

1- Did you receive information related to the care of a premature baby during pregnancy?

☐ Yes ☐ No

If yes, the source of information was from **(possible to select more than one answer)**:

a. Doctor b. Nurse/midwife c. Mass media d. Relative/friend

2- Did you receive information related to the care of the premature after delivery of this baby?

☐ Yes ☐ No

If yes, the source of information was from **(possible to select more than one answer)**:

a. Doctor b. Nurse/midwife c. Mass media d. Relative/friend

If your answer for the previous 2 questions was no, skip the next question (Q 3)

3- Did you perceive the information you received as adequate? ☐ Yes ☐ No

Part V: Mother's knowledge regarding care of a premature baby (maximum score 30)

A	Thermoregulation	Yes	No
1.	The premature baby should be placed in a warm environment	/	
2.	The premature baby loses body heat rapidly if uncovered	/	
3.	Covering a premature baby's head helps in keeping the baby's temperature	/	
4.	Premature babies should be placed near open windows with air drafts		X
5.	A premature baby could be placed on cold beds or a blanket		X
6.	Premature babies should be manipulated frequently		X
7.	The Source of heat is essential to keep a baby's normal temperature	/	
8.	Handling the baby with warmed hands is necessary	/	
9.	Dressing the baby in previously warmed clothes is necessary	/	
10.	The baby should be bathed with warm water with immediate drying and covering after bathing	/	
B	Feeding		
1.	Any premature baby can tolerate breastfeeding		X
2.	A premature baby may be kept NPO	/	

3.	A premature baby may be given feeding through NGT	/	
4.	Feeding a premature baby through NGT is by pushing milk through a syringe in the tube		X
5.	Pacifiers may be used for NPO premature baby	/	
6.	Premature babies should receive small and frequent amounts of milk	/	
7.	Burping a premature baby during and after feeding is not essential		X
8.	Expressed breast milk for baby is best if unable to receive breastfeeding	/	
C	Phototherapy		
1.	The premature baby's temperature should be monitored during phototherapy	/	
2.	The premature baby's position should be changed frequently during phototherapy	/	
3.	The Baby's eyes should be covered during phototherapy	/	
4.	The Baby's genitalia should be covered during phototherapy	/	
5.	Baby under phototherapy should be kept well hydrated through IV fluid or milk feeding	/	
D	Infection & Skin care		
1.	The diaper can cover the umbilical cord		X
2.	The umbilical cord should be kept dry and clean	/	
3.	A soiled diaper should be changed immediately	/	
4.	Hand hygiene should be done before handling a premature baby	/	
5.	The umbilical cord should be covered		X
6.	Instruments used for the care of the baby could be shared with others		X
7.	Perineal and umbilical cord areas should be monitored closely for infection	/	

Scoring note. In Part V, "/" and "X" are scoring-key marks indicating the response option scored as correct for each item; they are not participant responses. In the English version, "/" marks items where "Yes" was scored as correct, and "X" marks items where "No" was scored as correct.

Interviewer-administered questionnaire (Arabic version)

رقم الاستبانة:.....................

أرجو الإجابة على الأسئلة التالية:

**أولاً: البيانات الديموغرافية والاجتماعية**	
**عمرك بالسنوات:**..............................سنة	1.
**مكان السكن (المحافظة):**..	2.
**عدد سنوات تعليمك:**..	3.
**عدد سنوات تعليم الزوج:**..	4.
متوسط الدخل الشهري بالشيقل:.......................	5.
**عمل أم الطفل:** 1- تعمل/موظفة 2- ربة منزل	6.
**عمل والد الطفل:** 1- يعمل/موظف 2- لا يعمل	7.
**ثانياً: عوامل متعلقة بالولادة**	

1.	**عوامل خطر عند الأم:** 1- السكر 2. ارتفاع ضغط دم الحمل 3- انفجار مبكر لأغشية الجنين 4- ولادة قيصرية 5- حمل بتوأم 6- أخرى (مع التحديد)..........................
2.	**طريقة ولادة الطفل:** 1- طبيعية مهبلياً 2- مهبلياً باستخدام شفاط 3- قيصرية
3.	عدد الأطفال المولودين خدج سابقاً (غير هذا الطفل): .... طفل
4.	عمر حمل هذا الطفل بالأسابيع:.....................أسبوع
5.	عدد الأطفال لديك (مع هذا الطفل):................. طفل
6.	**ترتيب هذا الطفل بين أطفالك:** 1- الأول 2- الثاني 3- الثالث 4- الرابع أو أكثر
7.	**جنس الطفل:** 1- ذكر 2- أنثى
8.	تاريخ ميلاد الطفل:............................
**ثالثاً: الخدمات الصحية ذات العلاقة بالأم**	
1.	عدد الزيارات لعيادة الحوامل خلال حملك بهذا الطفل:..................... زيارة
2.	**هل تلقيت متابعة خاصة بعيادة الحوامل متعلقة برعاية طفل خداج:** 1- نعم 2- لا
3.	هل تلقيت فحص من طبيب خلال متابعتك بعيادة الحوامل: 1- نعم 2 – لا
4.	هل تلقيت فحص من ممرضة/قابلة خلال متابعتك بعيادة الحوامل: 1- نعم 2 – لا
5.	هل أخبرك مقدم الخدمة الصحية عن كيفية الإستعداد لولادة طفل مبكراً (خداج): 1- نعم 2 – لا
**رابعاً: مصدر المعلومات**	
1.	**هل تلقيت معلومات ذات علاقة برعاية الطفل الخداج خلال فترة الحمل؟**<br>1- نعم 2 – لا<br>إذا كانت الإجابة بنعم، من كان مصدر تلك المعلومات (يمكنك اختيار أكثر من اجابة حسب ما ينطبق):<br>1- الطبيب 2- الممرضة/القابلة 3- الإعلام 4- أقارب/أصدقاء
2.	**هل تلقيت معلومات متعلقة برعاية الطفل الخداج بعد ولادة هذا الطفل:**<br>1- نعم 2 – لا<br>إذا كانت الإجابة بنعم، من كان مصدر تلك المعلومات (يمكنك اختيار أكثر من اجابة حسب ما ينطبق):<br>1- الطبيب 2- الممرضة/القابلة 3- الإعلام 4- أقارب/أصدقاء
3.	إذا كانت إجابتك للسؤالين السابقين ب (لا) لا تجيبي هذا السؤال:<br>هل تعتبري المعلومات التي تلقيتيها بخصوص رعاية الطفل الخداج كافية؟<br>1- نعم 2 – لا

خامساً: معلومات الأم حول العناية بالطفل الخداج

أ	الحفاظ على حرارة الطفل	صحيح	خطأ

1.	الطفل الخداج يجب وضعه في بيئة دافئة	/	
2.	الطفل الخداج يفقد حرارته بسرعة إن لم يغطى	/	
3.	تغطية رأس الطفل الخداج يساعد في الحفاظ على درجة حرارة جسمه	/	
4.	الطفل الخداج يتم وضعه بجانب الشبابيك المفتوحة التي يدخلها تيار هوائي		X
5.	يمكن وضع الطفل الخداج فوق سرير أو حرام بارد		X
6.	الأطفال الخدج يتم تحريكهم باستمرار		X
7.	مصدر للحرارة ضروري بجوار الطفل الخداج للحفاظ على حرارته	/	
8.	تتم ملامسة الطفل الخداج بأيدي دافئة	/	
9.	من الضروري الباس الطفل ملابس تم تدفئتها مسبقاً	/	
10.	تحميم الطفل الخداج يتم بماء دافيء مع سرعة التجفيف وتغطية الطفل بعد الحمام	/	
ب	تغذية الطفل	صحيح	خطأ
1.	أي طفل خداج يستطيع ممارسة الرضاعة من ثدي الأم		X
2.	الطفل الخداج من الممكن ألا يسمح له بأخذ أي شيء عبر الفم	/	
3.	الطفل الخداج من الممكن إعطائه الحليب عبر انبوب يصل للمعدة	/	
4.	تغذية الطفل عبر انبوب المعدة يتم من خلال دفع الحليب بواسطة سرنجة دفعاً في الانبوب		X
5.	البز الكاذب (اللهاية) يمكن استخدامه مع الطفل الخداج الذي لا يرضع عبر الفم	/	
6.	الطفل الخداج يتم إرضاعه بكميات صغيرة ومتكررة من الحليب	/	
7.	ليس من الضروري تكريع (تشجأة) الطفل الخداج أثناء وبعد الرضاعة		X
8.	يعتبر حليب الأم المشفوط أفضل للطفل في حال لا يستطيع الرضاعة من ثدي الأم	/	
ت	العلاج الضوئي	صحيح	خطأ
1.	يجب مراقبة حرارة الطفل الخداج أثناء وضعه تحت العلاج الضوئي	/	
2.	يجب أن يتم تغيير وضعية الطفل الخداج أثناء وضعه تحت العلاج الضوئي	/	
3.	يجب تغطية عيني الطفل الخداج أثناء وضعه تحت العلاج الضوئي	/	
4.	يجب تغطية الجهاز التناسلي للطفل الخداج أثناء وضعه تحت العلاج الضوئي	/	
5.	الأطفال تحت العلاج الضوئي يجب الحفاظ على السوائل لديهم من خلال اعطاء الحليب أو السوائل الوريدية	/	
ث	العدوى والعناية بالجلد	صحيح	خطأ
1.	الحبل السري يمكن تغطيته بالحفاظة		X
2.	يجب أن يحافظ على الحبل السري جافا ونظيفاً	/	
3.	الحفاظة المتسخة يجب تغييرها في الحال	/	
4.	غسل الأيدي بالصابون أو معقم ضروري قبل ملامسة الطفل	/	
5.	يجب تغطية الحبل السري		X
6.	الأدوات المستخدمة للطفل يمكن مشاركتها مع الآخرين		X

7.	المنطقة التناسلية و الحبل السري يجب مراقبتها باستمرار لعلامات العدوى	/	
